# A pilot study of AI-assisted reading of prostate MRI in Organized Prostate Cancer Testing

**DOI:** 10.2340/1651-226X.2024.40475

**Published:** 2024-10-29

**Authors:** Erik Thimansson, Sophia Zackrisson, Fredrik Jäderling, Max Alterbeck, Thomas Jiborn, Anders Bjartell, Jonas Wallström

**Affiliations:** a Department of Translational Medicine, Diagnostic Radiology, Lund University, Malmö, Sweden; b Department of Radiology, Helsingborg Hospital, Helsingborg, Sweden; c Department of Imaging and Functional Medicine, Skåne University Hospital, Malmö, Sweden; d Department of Radiology, Capio St Görans Hospital, Stockholm, Sweden; e Institution of Molecular Medicine and Surgery (MMK), Karolinska Institutet, Stockholm, Swedenl; f Department of Translational Medicine, Urological Cancers, Lund University, Malmö, Sweden; g Department of Urology, Skåne University Hospital, Malmö, Sweden; h Department of Urology, Helsingborg Hospital, Helsingborg, Sweden; i Department of Radiology, Institute of Clinical Sciences, Sahlgrenska Academy, University of Gothenburg, Sweden; j Sahlgrenska University Hospital, Gothenburg, Sweden

**Keywords:** Magnetic resonance imaging, prostatic neoplasms, artificial intelligence, prostate-specific antigen, overdiagnosis

## Abstract

**Objectives:**

To evaluate the feasibility of AI-assisted reading of prostate magnetic resonance imaging (MRI) in Organized Prostate cancer Testing (OPT).

**Methods:**

Retrospective cohort study including 57 men with elevated prostate-specific antigen (PSA) levels ≥3 µg/L that performed bi-parametric MRI in OPT. The results of a CE-marked deep learning (DL) algorithm for prostate MRI lesion detection were compared with assessments performed by on-site radiologists and reference radiologists. Per patient PI-RADS (Prostate Imaging-Reporting and Data System)/Likert scores were cross-tabulated and compared with biopsy outcomes, if performed. Positive MRI was defined as PI-RADS/Likert ≥4. Reader variability was assessed with weighted kappa scores.

**Results:**

The number of positive MRIs was 13 (23%), 8 (14%), and 29 (51%) for the local radiologists, expert consensus, and DL, respectively. Kappa scores were moderate for local radiologists versus expert consensus 0.55 (95% confidence interval [CI]: 0.37–0.74), slight for local radiologists versus DL 0.12 (95% CI: −0.07 to 0.32), and slight for expert consensus versus DL 0.17 (95% CI: −0.01 to 0.35). Out of 10 cases with biopsy proven prostate cancer with Gleason ≥3+4 the DL scored 7 as Likert ≥4.

**Interpretation:**

The Dl-algorithm showed low agreement with both local and expert radiologists. Training and validation of DL-algorithms in specific screening cohorts is essential before introduction in organized testing.

## Introduction

Screening for prostate cancer (PC) is attractive due to a long, organ-confined, asymptomatic stage, in contrast to an often incurable disease when symptomatic. The addition of MRI in a prostate-specific antigen (PSA)-based program reduces the overdiagnosis of indolent cancers [1]. Artificial intelligence (AI) models have the potential to support radiologists, possibly improving accuracy and efficiency and reducing variability [2]. However, the robustness and generalizability of AI models in a true clinical setting remain uncertain [3]. To our knowledge, no earlier studies have tested the performance of AI models in cohort-based organized testing for PC.

In December 2022, the Council of the European Union recommended evaluating the feasibility and effectiveness of organized prostate cancer testing (OPT) with PSA testing and MRI as a follow-up test to select men for a prostate biopsy [4]. National and international organized testing, coupled with the paradigm shift towards ‘MRI first’ (where MRI examination is performed before biopsies), will likely increase the number of prostate MRIs.

Our research group plans a retrospective central review study of approximately 400 prostate MRIs from regional OPT [5, 6], reported by local radiologists, with expert radiologists’ consensus as the reference standard. In the same study, several AI models will be evaluated. To optimize the design of this upcoming AI-model evaluation, we are conducting the current study as a feasibility test. The aim is to evaluate the performance of a commercially available AI model in a subset of the OPT cohort.

## Material and methods

### Study design and population

**Figure 1:**
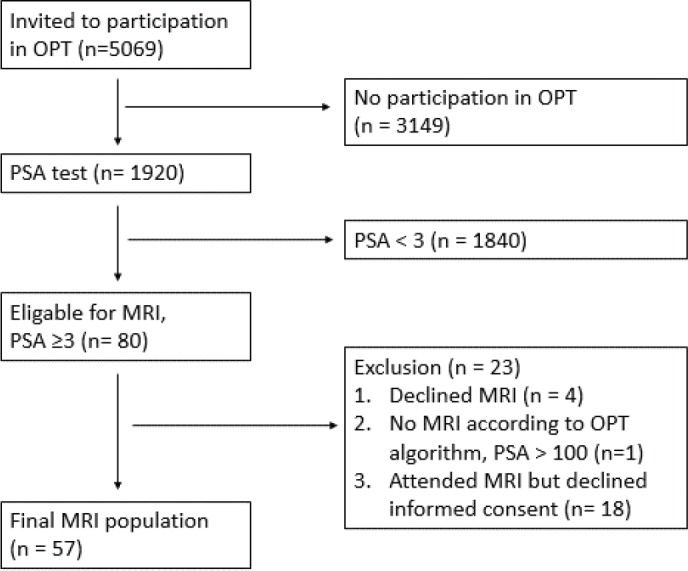
Study cohort.

This retrospective multicenter study was approved by the Swedish Ethical Review Authority (entry no. 2020-03923 and 2021-06647-02) with informed consent. The cohort has two subsets: (a) year 2020 OPT pilot study (*r*), 999 men, age 50 (*n* = 367), age 56 (*n* = 327) and age 62y (*n* = 305) who were randomly selected from 33 municipalities in the southern County of Sweden (Region Skåne, RS), and (b) year 2021 until 15/06/2021, all men aged 50 in RS (*n* = 4,070). Patient characteristics for the MRI population and biopsy population (age, prostate-specific antigen PSA, prostate volume PV and PSA density PSAD) are presented in Table 1. In total, 5,069 men were invited, 1,920/5,069 men participated and had a PSA test, 80/1,920 men had PSA > 3 µg/L, 75/80 men had an MRI and 57/75 men gave informed consent. Figure 1 shows the study cohort and final study population.

**Table 1:** Patient characteristics for the MRI population and biopsy population.

	Overall MRI population *N* = 57	Biopsy population *N* = 21
** **Age (year), median (IQR)	56 (50–62)	56 (50–62)
PSA (µg/L), median (IQR)	3.6 (3.2–5)	5 (3.5–6.8)
Prostate volume (mL), median (IQR)	40 (32–48)	35 (30–65)
PSAD local radiologist (µg/L/cm^3^), median (IQR)	0.095 (0.08–0.14)	0.14 (0.09–0.22)

MRI: magnetic resonance imaging; IQR: interquartile range; PSA: prostate-specific antigen; PSAD, prostate-specific antigen density.

### MRI technique and prostate MRI reporting

The MRI examinations were performed at eight radiology departments in RS with 10 scanners, four scanner models, one vendor, and 1.5 T and 3 T field strengths. The bi-parametric MRI (bpMRI) protocol was set up according to the Prostate Imaging-Reporting and Data System (PI-RADS) 2.1 document [7] and included T2-weighted imaging (T2W) in three planes and diffusion-weighted imaging (DWI) with calculated or acquired high b values (*b* = 1,500 s/mm^2^). MRI reading and reporting were standardized, and according to PI-RADS, reports included prostate volume calculation for PSA density, focal lesion characterization (PI-RADS 1–5), and localization (on a sector-based biopsy map). All MRIs were reported by the local OPT-associated radiologist. A central review of all MRI examinations was performed by two radiologists sub-specialized in prostate imaging (8 and 7 years of experience). The experts were blinded to clinical information and biopsy output, and assessments were conducted individually with consensus when needed. Additional characteristics regarding the DWI sequences used in the study are presented in Supplementary materials.

### Biopsy

**Figure 2:**
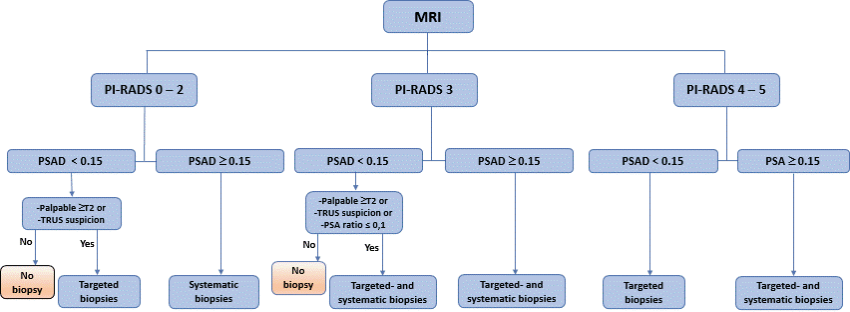
The Region Skåne OPT biopsy algorithm in force during the execution of the study.

Figure 2 shows the flowchart biopsy algorithm for OPT. Ultrasound-guided tran**s**rectal biopsies with cognitive or MRI fusion technique were performed on all PI-RADS ≥4 with the addition of systemic biopsies if PSAD ≥0.15 µg/L/cm^3^. PI-RADS 3 lesions had targeted and systemic biopsies if PSAD ≥0.15 µg/L/ cm^3^ and PI-RADS ≤2 had systemic biopsies if PSAD ≥0.15 µg/L/ cm^3^. Exceptions based on clinical assessment are outlined in the flowchart in Figure 2.

### AI model

**Figure 3:**
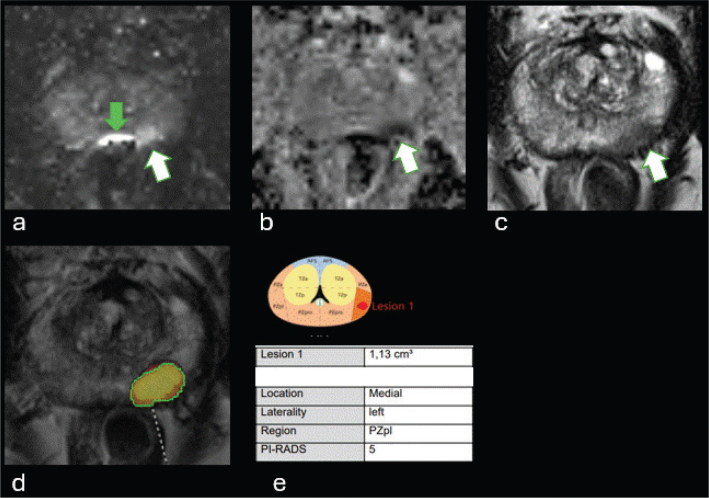
56 yo man, PSA 3.1 µg/L, PSAD 0,10 µg/L/cm^3^. MRI lesion dorsal portion PZ midgland, characterized as PI-RADS 4 by radiologist. (a) DWI b1500, white arrow indicates lesion, grey arrow artefact from rectal gas. (b) ADC, arrow indicates lesion. (c) T2W tra, arrow indicates lesion. (d) heatmap with lesion segmentation from DL algorithm, the lesion was characterized as Likert 5/PI-RADS 5. (e) lesion localization in sector map by DL-algorithm and lesion volume.

A commercial advanced viewing and visualization software for PI-RADS reporting with AI-based prostate lesion detection and classification (syngo.via MR Prostate AI, version VB50, Siemens Healthineers, Forchheim, Germany) was used for the present evaluation. The fully automated AI module consists of a pre-processing pipeline, a deep learning-based lesion detection, and a classification algorithm [8]. The preprocessing pipeline selects the T2W and DWI and from DWI computes a synthetic high *b*-value image at *b* = 2,000 s/mm^2^. Whole-gland segmentation is performed on T2W using a deep learning-based method [9]. After segmentation, a rigid registration is conducted to align the diffusion-weighted images to the T2W images. The DL algorithm then automatically detects cancer suspicious lesions and classifies each detected lesion using a Likert scale. The DL algorithm output consists of a heat map, three-dimensional lesion contours, and localization in the PI-RADS sector map. An example case with DL output is shown in Figure 3. In a clinical workflow, the radiologist would accept or reject the classification proposals from the DL algorithm. In this study, we instead translated the DL algorithm proposals without radiologist interpretation, Likert 4 and 5 was translated to PI-RADS categories 4 and 5, respectively (no cases were scored as overall Likert 3 lesions by the DL algorithm). The DL algorithm was not trained or exposed to any of the MRI data included in the study.

### Lesion fitting

An expert radiologist evaluated the location of all lesions from the DL algorithm and translated the localization from the PI-RADS sector map to the national sector map [10]. Lesions were considered matching if located in the same or adjacent ipsilateral sectors. The same method was used for fitting lesions between local radiologist and expert radiologist.

### Statistical analysis

All analyses were performed on a per-patient level. A positive MRI was defined as PI-RADS v2.1 assessment category 4–5 (radiologists) or Likert score 4–5 (deep learning [DL]). Significant PC was defined as Gleason score ≥7. Agreement between pairs of observers was evaluated with Cohens kappa with linear weights (slight agreement 0.01–0.20, fair agreement 0.21–0.40, moderate agreement 0.41–0.60, substantial agreement 0.61–0.80, and almost perfect agreement 0.80 to 1). All analyses were performed using SPSS (version 29).

## Results

A total of 57 men performed MRI. Twelve local radiologists with varying experience (1–12 years in prostate MRI reporting) reported the cases in the study. The number of positive MRIs with overall PI-RADS of 4–5 was 13 (23%), 8 (14%), and 29 (51%) for the local radiologists, expert consensus, and DL, respectively. The corresponding number of negative MRIs with PI-RADS scores of 1–2 were 31 (54%), 39 (68%), and 28 (49%). The corresponding number of examinations with an overall PI-RADS score of 3 was 13 (23%), 10 (18%), and 0 (0%). The PI-RADS score distribution is shown in Table 2.

**Table 2:** The PI-RADS/Likert score distribution.

	Local radiologist	Expert consensus	DL
PI-RADS/Likert 5, *n* (%)	1 (2)	1 (2)	8 (14)
PI-RADS/Likert 4, *n* (%)	12 (21)	7 (12)	21 (37)
PI-RADS/Likert 3, *n* (%)	13 (23)	10 (18)	0
PI-RADS/Likert ≤2, *n* (%)	31 (54)	39 (68)	28 (49)

DL: deep learning; PI-RADS: Prostate Imaging-Reporting and Data System..

The DL was concordant with the expert consensus in 26 out of 57 cases (46%) and with the local radiologists in 23 out 57 (40%) cases. The expert consensus was concordant with the local radiologist in 39 out of 57 cases (68%). In Table 3, cross-tabulations of PI-RADS scores are shown.

**Table 3a:** PI-RADS/Likert crosstabulation DL versus expert consensus.

Expert consensus	DL				
	2	3	4	5	Total
2	22	0	12	5	39
3	4	0	6	0	10
4	2	0	3	2	7
5	0	0	0	1	1
Total	28	0	21	8	57

DL: deep learning.

**Table 3b:** PI-RADS/Likert crosstabulation DL versus local radiologist.

Local radiologist	DL				
	2	3	4	5	Total
2	17	0	10	4	31
3	6	0	6	1	13
4	5	0	5	2	12
5	0	0	0	1	1
Total	28	0	21	8	57

DL: deep learning.

**Table 3c:** PI-RADS crosstabulation local radiologist versus expert consensus.

Local radiologist	Expert consensus				
	2	3	4	5	Total
2	29	2	0	0	31
3	7	4	2	0	13
4	3	4	5	0	12
5	0	0	0	1	1
Total	39	10	7	1	57

The agreement according to Cohen’s weighted Kappa with linear weights was moderate for local radiologists versus expert consensus 0.55 (95% CI: 0.37–0.74), slight for local radiologists versus DL 0.12 (95% CI: −0.07 to 0.32), and slight for expert consensus versus DL 0.17 (95% CI: −0.01 to 0.35).

A total of 21 biopsy procedures were performed based on the local radiologists’ assessment, 17 were targeted, and 4 were systematic due to a high PSA density of >0.15 µg/L/cm^3^ with PI-RADS ≤3. Biopsy outcomes are shown in Table 4.

**Table 4:** Biopsy outcomes.

Gleason score	Radiologist Index lesion PI-RADS	EXPERT Index lesion PI-RADS	DL Index lesion LIKERT	DL Tumor volume (mL)
3+3	4	3	4	0.35
3+3	4	3	0	-
3+3	4	4	4	0.46
3+4	4	4	0	-
3+4	3	4	4	0.51
3+4	3	3	0	-
3+4	2	2	5	0.35
3+4	4	3	4	0.97
3+4	2	2	0	-
3+4	3	4	4	0.05
3+4	4	4	5	1.13
3+5	4	3	4	0.13
4+5	5	5	5	1.44
benign	2	2	0	-
benign	4	2	4	0.13
benign	4	4	5	1.48
benign	4	2	0	-
benign	4	2	0	-
benign	3	2	0	-
benign	4	4	0	-
benign	2	2	4	0.05

DL: deep learning; PI-RADS: Prostate Imaging-Reporting and Data System.

With the OPT biopsy algorithm (Figure 2), the local radiologists’ assessment resulted in the detection of 8 GS ≥7 PC with targeted biopsy and 2 GS ≥7 PC with systematic biopsy only. In addition, 3 GS 6 PC were detected with targeted biopsy and none with systematic biopsy.

Based on the local radiologist assessment, 36 men were not biopsied. Nine had PI-RADS 3 with PSA density below the threshold (<0.15 µg/L/cm^3^) and the remaining 27 had PI-RADS 2. In comparison, the DL scored 17 out of these 36 men as PI-RADS ≥ 4 and the expert consensus scored none as PI-RADS ≥ 4.

## Discussion

In this study, we evaluate a commercially available DL algorithm for lesion detection and characterization in OPT.

The agreement between DL and expert radiologists was low, with 40% agreement for PI-RADS scoring and a Kappa score of 0.17. In particular, the DL scored three times as many cases as moderately to highly suspicious for significant PC, PI-RADS ≥4, compared to the expert consensus, 29 versus eight cases, and more than twofold for DL versus local radiologist, 29 versus 13 cases.

Few studies have evaluated DL algorithms in a screening setting but similar to our approach Winkel [11] studied performance of a DL algorithm compared to two experienced radiologists in a screened cohort of 48 men with a Kappa score of 0.42 between DL and expert. In a nonscreening cohort, Sanford et al. [12] assessed inter-reader agreement with an expert radiologist. The authors concluded that the AI model demonstrated consistency in predicting high-risk lesions (approximately 55% of PI-RADS 4 and 80% of PI-RADS 5) and had similar agreement to the radiologist, with κ scores of 0.4 versus 0.34. Schelb et al. [13] evaluated a DL algorithm in a study with 62 patients from a nonscreening cohort where 26 had significant PC. The authors reported similar performance for a DL algorithm compared to eight radiologists with at least 3 years of experience in reporting prostate MRI. Thus, we report a lower inter-reader agreement between expert radiologist and DL algorithm compared to previous studies, which is likely explained by the high frequency of unsuspicious MRIs in our cohort. It is important to note that the DL algorithm was not trained in a screening cohort, whereas the two experts are highly experienced in reading prostate MRI in screening/organized testing scenarios. According to our clinical experience, a different approach is needed when reviewing prostate MRI in a screening cohort with younger men compared to the typically older patient with clinical suspicion of PC.

The agreement between local radiologists and expert consensus was moderate, the local radiologists scored more cases as highly suspicious, 13 versus eight cases, but clearly fewer than the DL. Several previous studies have reported moderate agreement between radiologists in PI-RADS scoring [14, 15]. Quality assessment of reporting, continual biopsy feedback, and AI have been proposed as ways of reducing variability [16].

In the OPT diagnostic algorithm, a positive MRI with PI-RADS 4–5 or a negative MRI with PI-RADS 1–3 and a PSA density of 0.15 µg/L/cm^3^ or higher results in referral to the urologist for biopsy and consultation. Thus, any shift in diagnostic accuracy directly impacts the outcomes of OPT. Although the DL showed promising results for cancer detection, the high rate of highly suspicious findings among cases scored as negative by the expert groups is problematic. Out of 35 men with a negative MRI according to the local radiologist assessment, notably the DL algorithm scored 17 as highly suspicious and the expert consensus scored none. Although most of these men were not biopsied, it would be highly unlikely that a majority had significant PC based on the low prevalence of disease in 50-year-old men and the high negative predictive value of expert radiologists in screening [1].

In our MRI cohort, 21/57 (37%) men were selected for biopsy based on local radiologists’ assessment in combination with the OPT algorithm. In the scenario that the DL algorithm served as input to the OPT biopsy algorithm, more than half of the men (31/57, 54%) would have been selected for biopsy. In the scenario that the expert groups served as input, only eight men would be selected for biopsy based on PI-RADS.

One important potential use of DL algorithms in screening is to ‘rule out’ unsuspicious MRIs as the prevalence of significant PC is lower compared to clinical cohorts. As reference, the screening studies G2 and STHLM-3 MRI reported around two-thirds of negative MRI [17] using only expert radiologists. With a threshold of PI-RADS 3/Likert 3 the DL algorithm scored substantially fewer MRIs as negative, (49%) compared to expert radiologists (85%) and local radiologist (77%). In the scenario of using the evaluated AI model for ‘rule out’ with threshold PI-RADS ≤3, approximately half of the men would be ‘ruled out, notably including two men with biopsy proven intermediate risk PC.

This highlights the challenge in implementing AI models; in both the clinical and in the screening context prostate MRI is intended primarily to add value by lowering the number of men selected for biopsies and by reducing overdiagnosis of low-risk PC. It is therefore crucial that any AI models implemented do not significantly increase the number of men selected for biopsies. AI models intended for PC screening will need to be trained and validated in multicenter screening MRI datasets to show the desired results. In addition, the performance of radiologists working with input from AI models in a clinical setting should be evaluated. Based on the conclusions drawn from the current study, a larger-scale MRI review study is planned, encompassing eight times the number of MRIs, to evaluate the performance of various AI models and their potential added value within OPT.

## Limitations

This is a feasibility study with a limited number of participants. Biopsy was performed according to OPT guidelines. The tested DL algorithm was not trained to perform optimally in a screening cohort consisting of younger men with smaller and denser prostates and with a lower prevalence of significant PC. The implementation of a DL algorithm in the clinical workflow would likely involve a somewhat different use compared to how the algorithm was applied in this pilot study. Future prospective studies will need to address how to optimally implement DL algorithms in day-to-day clinical practice.

## Conclusion

A deep learning algorithm trained for clinical prostate MRI showed low agreement with both local and expert radiologists in a in a pilot cohort with elevated PSA levels in OPT. Training and validation of deep learning algorithms in specific screening cohorts are essential before introduction in organized testing.

## Supplementary Material

A pilot study of AI-assisted reading of prostate MRI in Organized Prostate Cancer Testing

## Data Availability

Image data and outcome data from biopsies are protected in accordance with the ethical approval for the study. The algorithm evaluated in the study is patent protected, and therefore, the coding is not open source.
